# Hematological reference intervals for Danish crossbred Landrace Yorkshire Duroc (LYD) pigs used in biomedical research

**DOI:** 10.1186/s13028-025-00798-6

**Published:** 2025-02-24

**Authors:** Kirstine Færgemand Præstegaard, Anne Winther-Larsen, Birgitte Saima Kousholt

**Affiliations:** 1 Department of Clinical Medicine, AUGUST, Aarhus University, Palle Juul-Jensens Boulevard 99, 8200 Aarhus N, Denmark; 2 Department of Clinical Biochemistry, Aarhus University Hospital, Palle Juul-Jensens, Boulevard 99, 8200 Aarhus N, Denmark

**Keywords:** Idexx ProCyte, Porcine hematology, 3Rs, Refinement

## Abstract

**Background:**

The health and welfare of pigs used in biomedical research is essential to research quality and compliance with the 3Rs (replacement, reduction, and refinement). Hematological variables are objective markers to quantitatively determine health issues and evaluate physiological differences before and after experimental procedures. There are no recent validated hematologic reference intervals (RIs) published for Danish crossbred Landrace Yorkshire Duroc (LYD) pigs to aid researchers and veterinarians in their decision-making. The objective of this study was to establish hematologic RIs for LYD pigs used for biomedical research. Blood samples were collected from healthy female LYD pigs (35–65 kg) and analyzed using the in-house ProCyte Dx Hematology Analyzer. Means with 90% confidence intervals for lower and upper limits were calculated according to guidelines by the American Society of Veterinary Clinical Pathology.

**Results:**

Inspection of 141 pigs led to 133 blood samples available for analyses after exclusions due to clinical signs of disease, inadequate tube filling or presence of macroscopic clots. Thirty-two samples reported platelet abnormalities and upon further investigation these samples were excluded when calculating RIs for platelets and platelet indices. Other measurements were not affected. The RI for red blood cells, hemoglobin, hematocrit and white blood cells were 5.10–7.00 × 10^6^/µL, 9.36–12.29 g/dL, 30.46–40.47%, and 11.73–25.00 × 10^3^/µL, respectively.

**Conclusions:**

Our study provides RIs for hematological variables in LYD pigs, revealing significant differences from published RIs of other breeds. These findings highlight the influence of factors like age, breed and health status on measurements, emphasizing the importance of using breed-specific RIs. This research supports the 3Rs, guiding better animal care and enhancing overall research quality.

**Supplementary Information:**

The online version contains supplementary material available at 10.1186/s13028-025-00798-6.

## Background

The pig is extensively used in biomedical research and for surgical training purposes due to the comparative nature of its organs [1]. In the European Union and Norway, approximately 73,500 pigs were utilized for scientific purposes in 2020, with global figures estimated to be significantly higher [2].

Ensuring the health and welfare of laboratory pigs is essential not only for the quality and consistency of research outcomes but also to adhere to the principles of the 3Rs—replacement, reduction, and refinement—governing the ethical use of animals in research [3, 4]. Clinical laboratories play an indispensable role in this context, providing objective data that assist in the evaluation, monitoring, and decision-making processes of an animal’s fate. Reliable and accurate RIs for laboratory analyses are crucial to quantitatively and unbiased identify health issues, make informed decisions on the inclusion or exclusion of animals, and assess physiological changes before and after experimental procedures, [5, 6]. These RIs support the 3Rs by facilitating reduction—using the fewest animals necessary—and refinement—minimizing or eliminating pain and distress while enhancing welfare during experiments.

In Denmark, the production of Danish crossbred LYD pigs exceeds 30 million annually, with 14 million exported and approximately 24,000 used for scientific purposes within the country [7, 8]. Despite widespread use in research, information on up-to-date hematologic RIs according to guidelines seems to be missing or appear outdated in LYD crossbred pigs. While hematologic reference values are available for miniature pigs [9–13] and for other breeds of domestic pigs [14–25], these can only be applied cautiously due to potential differences in breed, age, and gender [26]. To date, only one recent study describes hematologic RIs in LYD sows at mid-gestation [27]. In research, however, juvenile animals are primarily used and hematologic RIs of gestating animals is not applicable. No recent updated and validated hematologic RIs for LYD pigs are retrievable in the literature. Hence, benchmarking new RIs is warranted.

The objective of this study was to establish hematologic reference values according to established guidelines for healthy LYD pigs used for biomedical research or surgical training purposes.

## Methods

This study was conducted from March 2022 until November 2022 at the Department of Clinical Medicine at Aarhus University. Blood samples were obtained from pigs used for surgical training courses approved by The Animal Experimental Council in Denmark, license number 2021-15-0201-00966. The study protocol for blood sampling followed the guidelines of the American Society of Veterinary Clinical Pathology (ASVCP), the International Federation of Clinical Chemistry (IFCC), and the Clinical and Laboratory Standards Institute (CLSI) on the de novo establishment of RIs [28–31].

### Animals

All pigs (Sus scrofa domesticus) included were female gender and crossbred Landrace × Yorkshire × Duroc breed. They were retrieved from a farm that screens for seven specific pathogens as part of a national farm health-monitoring program (SPF) [32]. At day four of age, pigs received an intramuscular injection of gleptoferron combined with the anticoccidial toltrazuril for the simultaneous prevention of piglet coccidiosis and iron deficiency anemia (Forceris, CEVA Animal Health A/S, Vejle, Denmark). Pigs were additionally vaccinated for porcine circovirus (Ingelvac CircoFLEX, Boehringer Ingelheim Animal Health Nordics A/S, Copenhagen, Denmark) and for lawsonia intracellularis (Porcilis Lawsonia ID Vet., MSD Animal Health A/S, Copenhagen, Denmark). Upon arrival on the research farm, the pigs were housed in groups according to the Institution’s Animal Welfare Policy that complies with national legislation on animal experimentation. Housing has been described previously [33]. Briefly, pigs are housed indoors with photoperiods 12:12 h light:dark, and room temperature at 20 ± 4 ℃. Straw bedding is used in all pens and sprinklers are available to moist the pens if needed. Pigs are fed with a standard diet with extra fibres twice daily according to weight and have free access to water and hay.

### Selection of reference pigs

The pigs were examined twice for clinical signs of disease. The day after arrival, the pigs were visually inspected by a veterinarian at rest and in locomotion. The weight of the pigs was measured, and an identification number was given to each pig. Inclusion criteria were as follows: pigs weighing between 35 and 65 kg (estimated age range 3–4 months), bright, alert, and responsive, normal skin color, body condition, and activity. Exclusion criteria were any visible health concerns such as infected skin lesions, abscesses, tail bites, diarrhea, coughing, or abnormal gait/lameness.

**Fig. 1:**
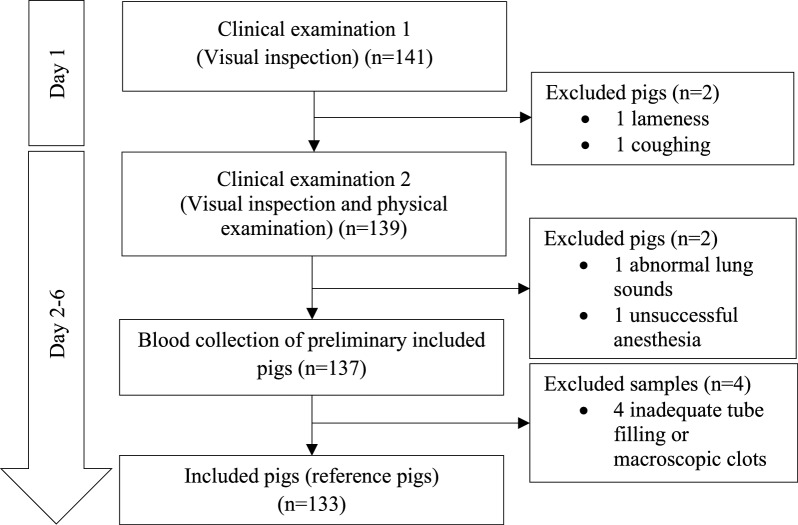
Study selection process of reference pigs for reference interval determination

On days 2–6, included pigs were subjected to an additional clinical examination that comprised a visual inspection at rest and in locomotion, and a physical examination after deep sedation by intramuscular injection of a solution of 1 mL/10 kg bodyweight of Zoletil (1.56 mg/kg Zoletil 50 Vet, Virbac, Denmark) dissolved in 1.56 mg/kg ketamine (Ketaminol Vet 100 mg/mL, Intervet, Denmark), 1.56 mg/kg xylazine (Rompun Vet 20 mg/mL, Bayer, Denmark) and 2.5 mg/kg butorphanol (Torbugesic Vet 10 mg/mL, Zoetis, Denmark). Measurements of body temperature, heart rate and rhythm, respiratory frequency and lung sounds were then obtained. Pigs were excluded if any of the following were present: body temperature above 40 degrees Celsius, arrhythmia, or abnormal lung sounds. Just after the examination, blood samples for RI determination were obtained from all included pigs. The selection process is presented in Fig. 1.

### Blood collection and handling

A tourniquet was positioned at the base of the ear and the skin disinfected. Venipuncture was performed in the left or right marginal ear vein by use of an intravenous cannula (BD Venflon Pro, 18GA 1.1 × 32 mm, Becton Dickinson Infusion Therapy, Helsingborg, Sweden) connected to a blood tube holder (Vacuette, Holdex, Single-Use Holder PP, Greiner Bio-One, Kremsmünster, Austria). Blood samples for hematological analysis were collected into 2 mL tubes containing ethylenediamine tetraacetic acid (EDTA) (BD Vacutainer®, K2EDTA 3.6 mg, 2 mL 13 × 75 mm, Becton, Dickinson and Company, Franklin Lakes, NJ, USA). Immediately after collection, tubes were gently inverted 10 times to ensure thorough mixing of the additive with the blood. Incorrectly filled tubes or tubes with macroscopic clots were excluded. All samples were identified and transported to the university research laboratory while stored at room temperature. The median time between sample collection and analysis was 2:39 h (ranging from 1:30 to 4:54 h). A previously described semi-quantitative method of scoring, ranging from 1–4 (representing low to high difficulty in filling the tube), was used to evaluate sampling [34].

### Hematological analyses

After being inverted 10 times, blood samples were analyzed using the in-house Procyte Dx Hematology Analyzer (IDEXX Laboratories, Inc., Westbrook, Maine 04092, USA) with a species-specific setting for pigs. Complete blood cell counts (CBCs) included red blood cell (RBC) count, hemoglobin (HGB), hematocrit (HCT), mean corpuscular volume (MCV), mean corpuscular hemoglobin (MCH), mean corpuscular hemoglobin concentration (MCHC), reticulocyte count (absolute count and percentages), RBC distribution width standard deviation (RDW-SD), RBC distribution width as a coefficient of variation (RDW-CV), white blood cell (WBC) count, and the leukocyte differential count (absolute count and percentages). All samples were analyzed in duplicates and the average value of the duplicates was used. Blood films from each EDTA blood sample were prepared, air-dried, stained with Hemacolor (Hemacolor® Rapid staining of blood smear, Merck KGaA, 64,271 Darmstadt, Germany), fixed and stored. Blood films were examined by a trained medical laboratory technician in a blinded manner. The number of single platelets and platelets in clumps were calculated in eight high power fields and an estimated error in the total platelet count was calculated as "number of platelets in clumps"/"total counted number of platelets" (Additional file 1). Data were extracted from the Procyte Dx Hematology Analyzer and loaded into Excel (MS office, version 2016, Microsoft Corp., USA).

To ensure the stability of the analyzer, quality control was performed daily according to the manufacturer’s instructions. The mean value, standard deviation (SD) and the coefficient of variation (CV) of the quality controls are shown in an additional file (Additional file 2).

### Statistical analysis

Reference intervals were determined using the add-in software Analyze-It for statistical analyses in Microsoft excel (Analyze-It Version 3.0; Analyze-It Software, Leeds, UK). The data distribution of each hematological variable was assessed by visual inspection (q-q plots and histograms) and by the Shapiro–Wilks test with a significance level of 5%. Data showing a non-Gaussian distribution were transformed, if possible. Outliers were detected by the 1.5 interquartile range (IQR) method and removed [5, 31]. For Gaussian variables, the 2.5th, 50th and 97.5th percentiles with 90% confidence intervals (CIs) were estimated using parametric statistics (normal quantile) as recommended by the ASVCP for de novo determination of RIs in veterinary species [31, 35]. Variables with a non-Gaussian distribution were calculated non-parametrically (bootstrap quantile method) and the 2.5th, 50th and 97.5th percentiles with 90% confidence intervals were estimated.

## Results

A total of 141 pigs were inspected. Four pigs were excluded due to lameness (1), coughing (1), abnormal lung sounds (1), or unsuccessful anesthesia (1). Four blood samples were excluded before analysis because of inadequate tube filling (3) or presence of macroscopic clots (1). Thus, 133 blood specimens were available for RI determination (Fig. 1). Outliers, sample sizes and RIs with 90% CIs for lower and upper limits are shown in Table 1. Baseline variables of all included pigs are presented in Table 2. The data distribution for hematological parameters is visualized in Additional file 3.

**Table 1:** Hematologic reference intervals for LYD pigs, 35–65 kg

Analyte	n	outliers	Mean	SD	Median	Min	Max	Distribution	Method	LRL	URL	CI 90% of LRL	CI 90% of URL
RBC (M/µL)	133	0	6.05	0.48	6.08	4.97	7.31	Gaussian	Normal quantile	5.10	7.00	4.98–5.22	6.88–7.12
Hemoglobin (g/dL)	133	0	10.83	0.745	10.85	9.20	12.75	Gaussian	Normal quantile	9.36	12.29	9.18–9.55	12.11–12.47
HCT (%)	133	0	35.2	2.554	35.2	29.8	41	Gaussian	Normal quantile	30.46	40.47	29.92–31.00	39.76–41.19
MCV (fL)	133	0	58.27	2.701	58.45	52.15	63.95	Gaussian	Normal quantile	52.97	63.57	52.31–53.63	62.92–64.23
MCH (pg)	132	1	17.91	0.788	17.95	16.15	19.5	Gaussian	Normal quantile	16.36	19.45	16.17–16.55	19.26–19.65
MCHC (g/dL)	130	3	30.76	0.561	30.75	29.2	31.9	Gaussian	Normal quantile	29.66	31.86	29.52–29.80	31.72–32.00
Reticulocyte (K/µL)	130	3	40.22	13.85	38.6	13.35	81.75	Gaussian	Logarithmic transformation	19.19	74.95	17.61–20.90	68.81–81.65
Reticulocyte (%)	132	1	0.678	0.248	0.638	0.27	1.51	Gaussian	Logarithmic transformation	0.32	1.28	0.289–0.344	1.177–1.402
RDW-SD (fL)	132	1	37.07	1.922	36.9	32.05	42.55	Gaussian	Logarithmic transformation	33.46	40.96	33.04–33.88	40.44–41.48
RDW-CV (%)	133	0	20.49	1.49	20.75	16.95	24.4	Non-Gaussian	Bootstrap	17.52	23.47	17.11–17.89	22.63–24.31
WBC (K/µL)	132	1	18.368	3.378	17.878	10.885	27.64	Gaussian	Normal quantile	11.73	25.00	10.90–12.56	24.18–25.83
Neutrophil (K/µL)	130	3	5.731	1.825	5.425	2.865	12.825	Gaussian	Exponential function	3.17	10.45	2.96–3.39	9.37–11.91
Lymphocyte (K/µL)	133	0	11.274	2.112	11.245	6.02	17.075	Gaussian	Normal quantile	7.13	15.42	6.61–7.64	14.90–15.94
Monocyte (K/µL)	132	1	1.221	0.497	1.088	0.67	3.97	Gaussian	Exponential function	0.71	2.33	0.67–0.75	1.99–3.06
Eosinophil (K/µL)	131	2	0.175	0.198	0.13	0.01	1.395	Gaussian	Logarithmic transformation	0.01	0.80	0.01–0.02	0.62–1.02
Basophil (K/µL)	132	1	0.01	0.006	0.01	0	0.03	Non-Gaussian	Bootstrap	0.00	0.25	0.00–0.00	0.02–0.03
Neutrophil (%)	130	3	30.81	6.277	29.53	19.8	48.6	Gaussian	Exponential function	20.49	45.47	19.50–21.52	42.96–48.24
Lymphocyte (%)	132	1	61.693	6.765	61.85	46.8	79.85	Gaussian	Normal quantile	48.41	74.98	46.75–50.06	73.32–76.63
Monocyte (%)	133	0	6.642	2.348	6.1	3.6	18.1	Gaussian	Exponential function	3.92	12.45	3.67–4.13	10.80–15.50
Eosinophil (%)	127	6	0.808	0.656	0.7	0	3.1	Non-Gaussian	Bootstrap	0.09	2.78	0.02–0.1	2.28–3.08
Basophil (%)	132	1	0.061	0.043	0.05	0	0.15	Non-Gaussian	Bootstrap	0.00	0.14	0.00–0.00	0.10–0.15

*LYD* Landrace Yorkshire Duroc, *LRL* lower reference limit, *URL* upper reference limit, *RBC* red blood cells, *HCT* hematocrit, *MCV* mean corpuscular volume, *MCH* mean corpuscular hemoglobin, *MCHC* MCH concentration, *RDW-SD* red blood cell distribution width standard deviation, *RDW-CV* red blood cell distribution width as a coefficient of variation, *WBC* white blood cells

**Table 2:** Weight, temperature, pulse, respiration and SpO2 of reference pigs, estimated age 3–4 months

Variable					
	Weight (Kilogram)	Body temperature (°C)	Pulse (bpm*)	Respiration (bpm**)	SpO2 (%)
n	133	133	129#	128^†^	133
Mean ± SD	47,77 ± 9,63	38,93 ± 0,38	89,45 ± 16,00	52,80 ± 15,33	94,50 ± 2,72

*Beats per minute; **breaths per minute; SpO2, oxygen saturation; #four measurements missing; ^†^Five measurements missing

A “platelet abnormal distribution” flag was generated by the analyzer in 32 of the blood specimens. The degree of platelet aggregation in these samples was compared with a random sample of specimens without a flag, by examining blood films from the specimens. No difference in percent estimated error in the total platelet count was observed between blood films with and without platelet flagging. Furthermore, no correlation was found between sampling difficulty and samples with a flagging of platelets (Additional file 1).

The platelet flagging effect on the hematological parameters was evaluated by comparing RIs calculated with and without blood specimens with a flag for abnormal distribution of platelets. For RBCs and derived parameters and for WBCs variables, no effect was observed. For platelets (PLT-O and PLT-I) and to a lesser extend platelet indices (PDW, PPV-O, P-LCR and PCT), an effect could be seen reflected by a lowering of the lower reference limit if specimens with an abnormal platelet distribution were included in the RIs. Based on this investigation, the flagged specimens were excluded in the RI determination of platelet count and platelet indices but included in the determination of the remaining RIs.

## Discussion

The objective of this study was to establish hematologic reference values for commonly measured hematological parameters in healthy LYD pigs utilized for biomedical research or surgical training.

When we compared our findings to reference intervals of other porcine breeds such as the Norwegian grower pigs and juvenile Yorkshire pigs, slight variations were noticed [19, 25]. For instance, the RIs for red blood cells were RBC (5.10–7.00 × 10^6^/µL), HGB (9.36–12.29 g/dL), and HCT (30.46–40.47%) whereas the RIs published for Norwegian grower pigs were (6.4–8.8 × 10^6^/µL, 10.5–13.5 g/dL, and 0.34–0.44%, respectively) and for juvenile Yorkshire pigs (4.80–7.11 × 10^6^/µL, 8.20–11.7 g/dL, and 25.38–38.77%, respectively). The differences highlight the importance of establishing reference intervals for the specific breed and underscores the fact that there are variations in hematological variables among pigs of different breeds and ages [9–12, 14–25, 27]. In this regard it is worth noticing that the LYD pigs in this study were females and 3–4 months old based on weight. This life stage is characterized by rapid growth and significant physiological changes. Blood volume increases and the intake of essential nutrients, such as iron, is vital [26]. Notably, the RI for HGB in our study were significantly higher compared to piglets not treated with iron (3.56–7.74 g/dL) [16]. This reflects the iron supplementation the pigs in our study received and is consistent with previous findings of lower erythroid values in younger pigs without iron supplementation [36].

Comparison of RIs for WBC also revealed differences [14–17]. We noticed that the reference limits for WBC reported for Danish sows (Landrace-Yorkshire) at mid-gestation (8.67–23.64 × 10^9^/L) were lower compared to our findings. This finding highlights the importance of considering age and gestational status when interpreting hematological data [22]. Also, when comparing our results to small pig breeds, we noticed that these breeds had narrower RIs with lower limits, e.g., female miniature pigs (0–17.44 × 10^3^/L) and female minipigs (8.90–13.40 × 10^9^/L) [9, 13]. This may be due to genetic factors, but biosecurity and health status may also influence the results.

We thoroughly examined the health of the included pigs to ensure that the RIs generated were not inadvertently skewed by inclusion of sick animals [37]. WBC values in particular may vary considerably in the presence of some pathogens manifesting as inflammation and infection [37]. The two-step clinical examination included both observations at rest and during locomotion as well as temperature, respiratory and cardiovascular examinations. Hence, we are confident that we did not overlook clinical illness. We notice that some studies state that animals are deemed healthy upon appearance without further mentioning of clinical examinations. Other studies in particularly in pets describe a thorough examination process that also tests for common species-specific subclinical infections [34]. The animals in this study were part of a large national pathogen screening program and this further decreases the risk of including animals with subclinical disease.

Leukocytosis is also a common response to stress [26]. The stress associated with handling and restraint affects the leukogram [38, 39]. In this study, pigs were anesthetized during blood collection to minimize stress-related alterations in WBC parameters. Anesthesia itself may, however, also alter hematological parameters, thus there is no gold standard and one must always consider how the animals were handled during sampling [38].

We investigated female animals as this gender is used at the research facility. Gender influences RIs and must be kept in mind when interpreting these data. Sex hormones are e.g. known to influence erythropoiesis [40]. The animals in this study were, however, not sexually mature. It would be interesting to pursue this further and disclose if and how much difference there may be between genders at this life stage and whether it is necessary to divide RIs based on gender at such an early age. We notice that in 3-week-old Ontario nursing pigs representing both sexes, the reference interval is almost equivalent to our findings for WBC (93.0–136.0 g/L) [14–17]. Overall, when comparing our findings to other studies, the impact of age, breed, housing and husbandry, sexual maturity, gestation and disease on hematological variables must be considered.

A rigorous methodology was employed to ensure the accuracy of the generated RIs and avoid skewing and confounding factors. Pre-analytical factors such as the use of anticoagulants, sample collection and handling, and storage conditions can affect the integrity of blood samples [38, 41]. RIs were established using blood samples collected from the marginal auricular vein, as this site offers easy accessibility, is well-suited for catheterization without influencing preanalytical variability, and particularly advantageous for collecting small blood volumes. Proper needle selection is critical to maintain sample integrity and minimize preanalytical variability [42]. To ensure adequate blood flow and minimize shear stress, reduce the risk of hemolysis, platelet activation, and clot formation an 18-gauge needle was employed for blood collection in this study. Blood samples were collected in EDTA tubes according to the user manual for the dedicated hematology analyzer and advice from Idexx’ technical support. EDTA tubes effectively prevent clot formation by chelating calcium ions [41]. However, EDTA may induce platelet aggregation and potentially lead to pseudothrombocytopenia [41, 43–50]. Alternative tubes e.g., coated with citrate, theophylline, adenosine, and dipyridamole (CTAD) reduce platelet aggregation in feline samples and may offer similar benefits for porcine blood [34]. The use of CTAD tubes in the current hematology analyzer was strongly discouraged by Idexx. Observations from previous studies do however suggest that porcine plasma is hypercoagulable [51, 52]. Hence, it would be interesting to analyze porcine blood on an analyzer capable of analyzing blood collected in CTAD tubes. If this decreases the number of platelet flagging, it is worth considering if the ability to analyze CTAD tubes should be a prerequisite for hematological analyzers dedicated to analyses of porcine samples.

Hematological analyzers combine methods such as impedance and/or optical cell counting. Each method has its capabilities and limitations [53]. Impedance counters measure cell size and are affected by platelet aggregates. Optical cell counters utilize light scattering patterns that are less influenced by platelet aggregation but are, however, not immune to these errors [53]. The ProCyte Dx Haematology Analyzer used in this study combines laser flow cytometry, optical fluorescence, and laminar flow impedance. This combination provides more accurate separation between red blood cells and platelets and an enhanced WBC differential [54]. The analyzer software contains a “flagging system”, which indicates potential sources of errors in cellular identification. “Platelet abnormal distribution” was flagged in 32 out of 133 blood specimens. This flag indicates abnormal distribution of platelet size, shape, or number, or too few platelets available for accurate assessment. To investigate this further, we performed a microscopic evaluation of peripheral blood films as suggested in the Idexx Operators’ Guide [54]. We found no difference in percent estimated error in the total platelet count between blood films with and without platelet flagging. No trend was observed, and no correlation was found between sampling difficulty and samples with an abnormal distribution of platelets. Platelet aggregates can interfere with impedance counting by altering perceived cell size, leading to underestimation of platelet counts and a false increase in other cell types [53]. Platelet clumping has been reported to spuriously elevate leukocyte counts and influence specific leukocyte populations across species, e.g. eosinophils and neutrophils in dogs and basophils and eosinophils in cats [55–57]. Our study found no significant differences in erythrocyte or leukocyte values with and without abnormal platelet flagging, indicating that platelet aggregation specifically impacted platelet counts rather than other cell types. Recent advancements in automated platelet counting methods, based on fluorescent labeling and flow cytometry, provide a more accurate platelet count [58]. These technologies may represent the next step forward in veterinary hematology, potentially overcoming the challenges we experienced with platelet flagging. Nevertheless, our experience from this study highlights the importance of sampling collection techniques, and indeed the type of hematological analyzer used. There is however no doubt about the fact that the susceptibility to platelet aggregation in pigs is influenced by complex factors beyond sample handling and preparation. Future research should continue to explore this. Additionally, objective and standardizing methods for assessing platelet aggregation and investigating the behavior of porcine thrombocytes under various storage conditions will further enhance the accuracy and reliability of hematological assessments, ultimately improve the quality and applicability of the results.

## Conclusions

Our study discloses differences in reference values of several hematological parameters for LYD pigs in comparison to published RIs for other pigs, highlighting the influence of pre-analytical conditions, laboratory measurement techniques, and factors such as age, breed, gestation, and health status on hematological parameters. The findings emphasize the need for laboratories to establish laboratory-specific reference values for hematological parameters in pigs of different breeds and ages, adhering to stated pre-analytical procedures and using the available hematological analyzer. While our results may not perfectly translate to every pig breed, they offer a robust general reference that is useful to improve health assessments and physiological monitoring in both biomedical and veterinary contexts. Additionally, the LYD-specific RIs generated in this study serve as a vital tool for post-operative welfare monitoring and accurate interpretation of experimental outcomes. This step represents a significant 3R advancement toward refinement of experimental protocols and ethical use of animals in research. By providing these physiological standards, our work supports the broader aim of improving both scientific rigor and animal welfare in translational research and may find relevance in several settings such as disease modelling and xenotransplantation.

## Supplementary Information

**Additional file 1.** Estimated error (%) in the total platelet count. The number of single platelets and platelets in clumps were calculated in eight high power fields and an estimated error in the total platelet count was calculated as "number of platelets in clumps"/"total counted number of platelets".

**Additional file 2.** Quality controls. The mean value, standard deviation (SD) and the coefficient of variation (CV) of the quality controls.

**Additional file 3.** Scatter plots. The data distribution for hematological parameters.

## Data Availability

The datasets used and/or analyzed during the current study are available from the corresponding author on reasonable request.

## Declarations

### Prior publication

Data have not been published previously**.**
