# Supplementary material for: Hematological reference intervals for Danish crossbred Landrace Yorkshire Duroc (LYD) pigs used in biomedical research
Source: Acta Vet Scand. 2025 Feb 24;67:11. doi: 10.1186/s13028-025-00798-6 (PMC11853315; doi:10.1186/s13028-025-00798-6)
Supplement: Supplementary file 3 — Additional file 3. Scatter plots. The data distribution for hematological parameters. [file 13028_2025_798_MOESM3_ESM.docx]

**Figure 1:** Scatter plots for (A) RBC, red blood cells; (B) Hgb, hemoglobulin; (C) Hct, hematocrit; (D) MCV, mean corpuscular volume; (E) MCH, mean corpuscular hemoglobin; (F) MCHC, MCH concentration; (G) RDW-SD, red blood cell distribution width standard deviation; (H) RDW-CV, red blood cell distribution width as a coefficient of variation; (I) RET, Reticulocytes; (J) WBC, white blood cells; (K) NEUT, Neutrophils; (L) LYMPH, Lymphocytes; (M) MONO, Monocytes; (N) EO, Eosinophils.
